# Effect of repetitive transcranial magnetic stimulation on the ascending reticular activating system in a patient with disorder of consciousness: a case report

**DOI:** 10.1186/s12883-020-1607-9

**Published:** 2020-01-29

**Authors:** Sung Ho Jang, Young Hyeon Kwon

**Affiliations:** 1 Department of Physical Medicine and Rehabilitation, College of Medicine, Yeungnam University 317-1, Daemyungdong, Namku, Daegu, 705-717 Republic of Korea; 2 Department of Physical Medicine and Rehabilitation, College of Medicine, Yeungnam University 317-1, Daemyungdong, Namku, Daegu, 705-717 Republic of Korea

**Keywords:** Diffusion tensor tractography, Ascending reticular activating system, Consciousness, Repetitive transcranial magnetic stimulation

## Abstract

**Background:**

We report on a stroke patient with disorder of consciousness (DOC) who underwent repetitive transcranial magnetic stimulation (rTMS) and showed recovery of an injured upper ascending reticular activating system (ARAS) injury, which was demonstrated by using serial diffusion tensor tractography (DTT).

**Case presentation:**

A 45-year-old male patient was diagnosed as subarachnoid and intracerebral hemorrhages in the left fronto-parieto-temporal lobes. At 5 months after onset, the patient exhibited a persistent vegetative state, with a Coma Recovery Scale-Revised (CRS-R) score of 4. He underwent comprehensive rehabilitative therapy that included drugs for recovery of impaired consciousness and rTMS of the right dorsolateral prefrontal lobe. He recovered to a minimally conscious state (CRS-R: 13) at 7 months after onset and was transferred to a local rehabilitation hospital where he underwent similar rehabilitation but without rTMS. At 9 months after onset, his CRS-R score remained at 13. He was then readmitted to our hospital and underwent rehabilitation with rTMS until 10 months after onset. His CRS-R remained at 13, but his higher cognition had improved. The tract volume (TV) of the neural tract in the right prefrontal lobe in the upper ARAS on the 7-month DTT was higher than that on the 5-month DTT. However, compared to the 7-month DTT, the right prefrontal lobe TV was lower on the 9-month DTT. On the 10-month DTT, the TV of that neural tract had again increased.

**Conclusions:**

Increases in neural TV in the right prefrontal lobe of the upper ARAS that were associated with the periods of rTMS application were demonstrated in a stroke patient with DOC.

## Background

Consciousness is mainly controlled by actions of the ascending reticular activating system (ARAS). The introduction of diffusion tensor tractography (DTT), which is derived from diffusion tensor imaging (DTI) data, enables three-dimensional evaluation of the ARAS in a live human brain [1–4]. As a result, many studies have used DTT to show improvements in impaired consciousness concurrent with ARAS injury recovery in patients with disorder of consciousness (DOC) [5–9]. It has been widely reported that repetitive transcranial magnetic stimulation (rTMS) has a therapeutic effect on impaired consciousness in patients with DOC [10–12]; however, little has been reported on the direct effect of rTMS on the ARAS.

For patients with DOC, several brain stimulation therapeutics can be applied: deep brain stimulation, vagus nerve stimulation, rTMS, transcranial direct current stimulation (tDCS), or transcranial alternative current stimulation [13]. Among the non-invasive brain stimulation therapeutics, tDCS may be preferred over rTMS because the effects of tDCS on patients with DOC have been demonstrated in randomized controlled studies [11, 13]. In contrast, other studies have demonstrated the effects of rTMS in scores of patients with DOC [10, 12–14].

In this study, we report on a stroke patient with DOC who underwent rTMS and showed upper ARAS injury recovery, which was demonstrated by performing serial DTT.

## Case presentation

**Fig. 1:**
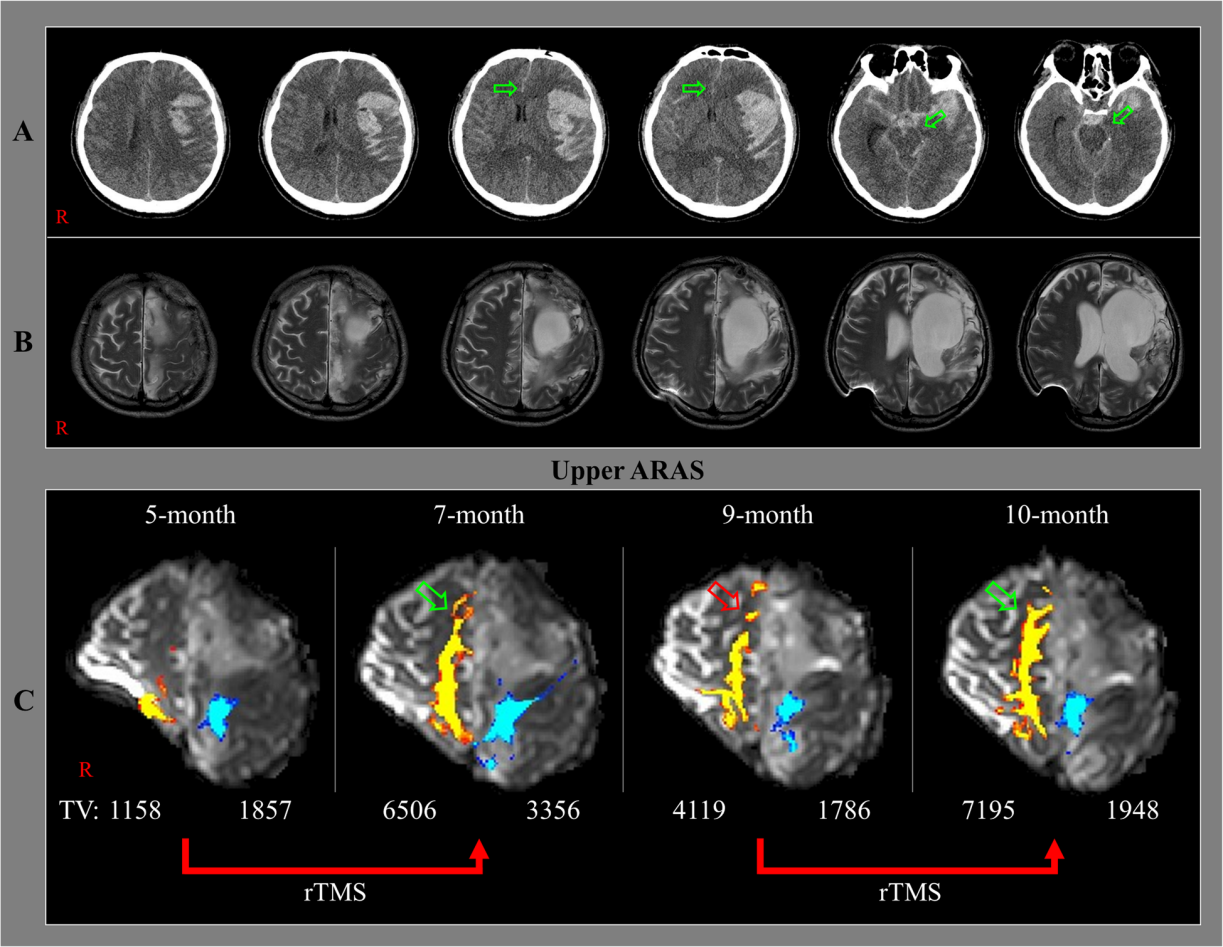
**a** Brain computed tomography images at onset show subfalcine and transtentorial herniations (arrows). **b** Brain magnetic resonance images at 5 months after onset show leukomalactic lesions in the left fronto-parieto-temporal lobes. **c** Results of diffusion tensor tractography (DTT) of the upper ascending reticular activating system (ARAS) of the patient. On the 7-month DTT, the neural tract of the right prefrontal lobe is increased (green arrow) compared to that on the 5-month DTT. However, the same neural tract is decreased (red arrow) on the 9-month DTT, but it is again increased (green arrow) on the 10-month DTT. ARAS: ascending reticular activating system, TV: tract volume, rTMS: repetitive transcranial magnetic stimulation

A 45-year-old male patient was diagnosed as subarachnoid and intracerebral hemorrhages in the left fronto-parieto-temporal lobes and underwent decompressive craniectomy with coiling of a ruptured aneurysm of the left middle cerebral artery at the neurosurgery department of a university hospital. Brain computed tomography showed subfalcine and transtentorial herniations (Fig. 1-A). At three months after onset, he was diagnosed as hydrocephalus and underwent ventriculoperitoneal (VP) shunting via the right parietal approach with cranioplasty. After five months after onset, he was transferred to the rehabilitation department of our university hospital. At that time, the patient exhibited a persistent vegetative state and had a Coma Recovery Scale-Revised (CRS-R) score of 4 (auditory function: 0, visual function: 0, motor function: 1, verbal function: 1, communication: 0, and arousal: 2) [15, 16]. Moreover, brain magnetic resonance images showed leukomalactic lesions in the left fronto-parieto-temporal lobes (Fig. 1b). At five months after onset, he began comprehensive rehabilitative therapy that included drugs for recovery of impaired consciousness (levodopa 750 mg, amantadine 200 mg, bromocriptine 10 mg, zolpidem 10 mg, and baclofen 75 mg), physical therapy, occupational therapy, and rTMS using a MagPro stimulator (Medtronic Functional Diagnostics, Skovlunde, Denmark) (Table 1). The right dorsolateral prefrontal lobe (Broadmann area 9) was subjected to rTMS at a frequency of 10 Hz with an 80% motor threshold intensity and 160 pulses over an 8-min period twice a day and with seven TMS sessions per week [17–19]. At seven months after onset (i.e., after 2 months of therapy), the patient recovered to a minimally conscious state (CRS-R score = 13; auditory function: 1, visual function: 3, motor function: 4, verbal function: 1, communication: 2, and arousal: 2). He was then transferred to a local rehabilitation hospital and underwent a reduced level of drug rehabilitation, with no rTMS, until 9 months after onset. His dopaminergic drug treatment was decreased due to aggravated spasticity (levodopa 500 mg, amantadine 150 mg, and bromocriptine 3.75 mg). After two months of such treatment, his CRS-R score (13) was unchanged. At nine months after onset, he was readmitted to our hospital and underwent similar drug treatments (same medications and doses) but also underwent rTMS. At 10 months after onset, although his CRS-R score remained at 13, his higher cognition indicators were improved; for example, he could blink his eyes the correct number of times when he was asked to blink his eyes as many times as the number presented. The patient’s wife provided signed and informed consent for participation in the study, and our institutional review board approved the study protocol.

**Table 1:** Changes in the patient’s rTMS application, CRS-R score, upper ARAS status, and medications

Duration from onset (months)	5 months	→ rTMS	7 months	→ No rTMS	9 months	→ rTMS	10 months
CRS-R	4		13		13		13 (higher cognition: improved)
Change of upper ARAS		Rt prefrontal lobe ↑		Rt prefrontal lobe ↓		Rt prefrontal lobe ↑	
Drugs		levodopa 750 mg, amantadine 200 mg, bromocriptine 10 mg, zolpidem 10 mg, baclofen 75 mg		levodopa 500 mg, amantadine 150 mg, bromocriptine 3.75 mg		levodopa 500 mg, amantadine 150 mg, bromocriptine 3.75 mg	

*rTMS* repetitive transcranial magnetic stimulation; *CRS-R* Coma Recovery Scale-Revised; *ARAS* ascending reticular activating system

### Diffusion tensor imaging

DTI scanning was performed four times (at five, seven, nine, and ten months after onset) using a 6-channel head coil on a 1.5 T Philips Gyroscan Intera system (Philips Healthcare, Best, Netherlands) programmed for single-shot, spin-echo, echo-planar imaging. Imaging parameters were as follows: acquisition matrix = 96 × 96; reconstructed to matrix = 192 × 192; field of view = 240 mm × 240 mm; TR = 10,398 ms; TE = 72 ms; parallel imaging reduction factor (SENSE factor) = 2; EPI factor = 59; b = 1000 s/mm^2^; and slice thickness = 2.5 mm. The Oxford Centre for Functional Magnetic Resonance Imaging of the Brain (FMRIB) software library was used to analyze the DTI data. Eddy current correction was applied to correct for head motion effects and image distortion. FMRIB diffusion software with the routines option (0.5 mm step lengths, 5000 streamline samples, curvature thresholds = 0.2) was used. For examination of the upper ARAS, the seed region of interest (ROI) was the intralaminar thalamic nucleus and the target ROI was the cerebral cortex [3]. Tract volume (TV) was determined for the upper ARAS.

On the 7-month post-onset DTT, theneural tract of the right prefrontal lobe in the upper ARAS was increased compared to that on the 5-month DTT. However, thesame neural tract was decreased on the 9-month DTT, but it was again increased on the 10-month DTT (Fig. 1b). Similarly, on the 7-month DTT, the TV of the same neural tract (TV = 6506) was higher than that on the 5-month DTT (TV = 1158). However, the TV of this neural tract was decreased on the 9-month DTT (TV = 4119), but it had again increased on the 10-month DTT (TV = 7195) (Fig. 1c).

## Discussion and conclusions

In this patient, we could not analyze the lower ARAS between the pontine reticular formation and the intralaminar thalamic nucleus or the right parietal portion of the upper ARAS due to artifacts related to the VP shunt. We suggest that the large lesions observed in the left cerebral cortex and subcortical white matter were the major cause of impaired consciousness in this patient [6–9, 20]. In addition, the right prefrontal injury due to subfalcine herniation may have contributed to impaired consciousness in this patient [21]. Although we could not analyze the lower ARAS in the brainstem due to artifact, and considering the brain computed tomography findings at onset, we think there is a possibility that the lower ARAS in the brainstem might have been injured by transtentorial herniation [22, 23].

In the current study, by using serial DTT, we were able to observe temporal changes in the upper ARAS of a stroke patient with DOC. The DTT results showed that the volume of the neural tract of the right prefrontal cortex increased in concert with the provision of comprehensive rehabilitation the included various medications and rTMS of the right prefrontal cortex between five and seven months after onset and at 9 months after onset. During this same period, the patient recovered from a persistent vegetative state (CRS-R = 4) to a minimally conscious state (CRS-R = 13) [15]. The TV, which indicates the total number of fibers in a neural tract, increased in the neural tract in the right prefrontal lobe (an important brain area associated with consciousness), suggesting it may be responsible for the impaired consciousness recovery in this patient [6–9, 20, 24]. During a two-month period, between seven and 9 months after onset, he underwent rehabilitation at a local rehabilitation hospital with reduced doses of dopaminergic drugs and without rTMS treatment. Although his consciousness was maintained at CRS-R = 13 at the end of that period, the TV of the neural tract in the right prefrontal cortex had decreased. Subsequently, the patient underwent similar drug rehabilitation (same medications and doses) in conjunction with rTMS on the right prefrontal lobe during a one-month period between nine and ten months after onset. As a result, the TV of the neural tract in the right prefrontal cortex increased to a level above that observed at 7 months after onset; in addition, indicators of the patient’s higher cognition level were improved. As a result, we suggest that rTMS application on the right prefrontal lobe was directly associated with improvements in the TV of the neural tract in the right prefrontal lobe. In conclusion, increases in neural TV in the right prefrontal lobe of the upper ARAS occurred in concert with the periods of application of rTMS in a stroke patient with DOC. Our results should be helpful when considering rehabilitation approaches for patients with DOC. In addition, DTT-based analysis of the ARAS can be useful in determining rehabilitation success in patients with DOC. However, some limitations of this study should be considered. First, DTT may underestimate fiber tract characteristics due to regions with fiber complexity and/or fiber crossing [25]. Second, because this is a case report, the study has limited generalizability. Especially, the response to rTMS can be different by some factors of individual patients [26, 27]. In addition, the rehabilitation hospital between 7 months after onset and 9 months after onset was different and the prescribed drugs which can affect consciousness were not same during this study (Table 1). Therefore, further studies involving a larger number of patients with DOC should be undertaken.

## Data Availability

The datasets generated and/or analyzed during the current study are not publicly available due to institutional restrictions but are available from the corresponding author on reasonable request.

## Abbreviations

ARAS: Ascending reticular activating system
CRS-R: Coma Recovery Scale-Revised
DOC: Disorder of consciousness
DTI: Diffusion tensor imaging
DTT: Diffusion tensor tractography
FMRIB: Functional Magnetic Resonance Imaging of the Brain
ROI: Region of interest
rTMS: Repetitive transcranial magnetic stimulation
tDCS: Transcranial direct current stimulation
TV: Tract volume
VP: Ventriculoperitoneal

## References

[1] Edlow BL, Takahashi E, Wu O, Benner T, Dai G, Bu L (2012). Neuroanatomic connectivity of the human ascending arousal system critical to consciousness and its disorders. J Neuropathol Exp Neurol.

[2] Yeo SS, Chang PH, Jang SH (2013). The ascending reticular activating system from pontine reticular formation to the thalamus in the human brain. Front Hum Neurosci.

[3] Jang SH, Lim HW, Yeo SS (2014). The neural connectivity of the intralaminar thalamic nuclei in the human brain: a diffusion tensor tractography study. Neurosci Lett.

[4] Jang SH, Kwon HG (2015). The ascending reticular activating system from pontine reticular formation to the hypothalamus in the human brain: a diffusion tensor imaging study. Neurosci Lett.

[5] Jang SH, Kim SH, Lim HW, Yeo SS (2015). Recovery of injured lower portion of the ascending reticular activating system in a patient with traumatic brain injury. Am J Phys Med Rehabil.

[6] Jang SH, Lee HD (2015). Ascending reticular activating system recovery in a patient with brain injury. Neurology.

[7] Jang SH, Chang CH, Jung YJ, Seo YS (2016). Change of ascending reticular activating system with recovery from vegetative state to minimally conscious state in a stroke patient. Medicine.

[8] Jang SH, Hyun YJ, Lee HD (2016). Recovery of consciousness and an injured ascending reticular activating system in a patient who survived cardiac arrest: a case report. Medicine.

[9] Jang S, Kim S, Lee H (2016). Recovery from vegetative state to minimally conscious state: a case report. Am J Phys Med Rehabil.

[10] Lefaucheur JP, Andre-Obadia N, Antal A, Ayache SS, Baeken C, Benninger DH (2014). Evidence-based guidelines on the therapeutic use of repetitive transcranial magnetic stimulation (rTMS). Clin Neurophysiol.

[11] Thibaut A, Bruno MA, Ledoux D, Demertzi A, Laureys S (2014). tDCS in patients with disorders of consciousness: sham-controlled randomized double-blind study. Neurology.

[12] Xia X, Bai Y, Zhou Y, Yang Y, Xu R, Gao X (2017). Effects of 10 Hz repetitive Transcranial magnetic stimulation of the left dorsolateral prefrontal cortex in disorders of consciousness. Front Neurol.

[13] Bourdillon P, Hermann B, Sitt JD, Naccache L (2019). Electromagnetic brain stimulation in patients with disorders of consciousness. Front Neurosci.

[14] Xie Y, Zhang T, Chen AC (2015). Repetitive transcranial magnetic stimulation for the recovery of stroke patients with disturbance of consciousness. Brain Stimul.

[15] Laureys S, Owen AM, Schiff ND (2004). Brain function in coma, vegetative state, and related disorders. Lancet Neurol.

[16] Giacino JT, Kalmar K, Whyte J (2004). The jfk coma recovery scale-revised: measurement characteristics and diagnostic utility. Arch Phys Med Rehabil.

[17] Elliott L, Walker L (2005). Rehabilitation interventions for vegetative and minimally conscious patients. Neuropsychol Rehabil.

[18] Kim YH, You SH, Ko MH, Park JW, Lee KH, Jang SH (2006). Repetitive transcranial magnetic stimulation-induced corticomotor excitability and associated motor skill acquisition in chronic stroke. Stroke.

[19] Georgiopoulos M, Katsakiori P, Kefalopoulou Z, Ellul J, Chroni E, Constantoyannis C (2010). Vegetative state and minimally conscious state: a review of the therapeutic interventions. Stereotact Funct Neurosurg.

[20] Laureys S, Faymonville ME, Luxen A, Lamy M, Franck G, Maquet P (2000). Restoration of thalamocortical connectivity after recovery from persistent vegetative state. Lancet.

[21] Jang SH, Kwon YH (2018). Injury of the lower ascending reticular activating system by subfalcine herniation in a patient with a cerebral infarct. Ann Rehabil Med.

[22] Jang SH, Chang CH, Jung YJ, Kim JH, Kwon YH (2019). Relationship between impaired consciousness and injury of ascending reticular activating system in patients with intracerebral hemorrhage. Stroke.

[23] Jang SH, Lim HW, Yeo SS (2015). Injury of the ascending reticular activating system by transtentorial herniation in a patient with intracerebral haemorrhage: a diffusion tensor tractography study. J Neurol Neurosurg Psychiatry.

[24] Boly M, Faymonville ME, Peigneux P, Lambermont B, Damas P, Del Fiore G (2004). Auditory processing in severely brain injured patients: differences between the minimally conscious state and the persistent vegetative state. Arch Neurol.

[25] Yamada K, Sakai K, Akazawa K, Yuen S, Nishimura T (2009). MR tractography: a review of its clinical applications. Magn Reson Med Sci.

[26] Chang WH, Uhm KE, Shin YI, Pascual-Leone A, Kim YH (2016). Factors influencing the response to high-frequency repetitive transcranial magnetic stimulation in patients with subacute stroke. Restor Neurol Neurosci.

[27] Lee J, Lee A, Kim H, Shin M, Yun SM, Jung Y (2019). Different brain connectivity between responders and nonresponders to dual-mode noninvasive brain stimulation over bilateral primary motor cortices in stroke patients. Neural Plast.

